# Role of gut microbiota in predicting chemotherapy-induced neutropenia duration in leukemia patients

**DOI:** 10.3389/fmicb.2025.1507336

**Published:** 2025-03-19

**Authors:** Yezi Huang, Lihong Liao, Yanjun Jiang, Si Tao, Duozhuang Tang

**Affiliations:** ^1^Department of Hematology, The Second Affiliated Hospital of Nanchang University, Nanchang, China; ^2^Jiangxi Provincial Key Laboratory of Hematological Diseases, Nanchang, China; ^3^Department of Oncology, The Second Affiliated Hospital of Nanchang University, Nanchang, China

**Keywords:** leukemia, gut microbiota, 16S rRNA, chemotherapy, neutropenia

## Abstract

**Background:**

Acute leukemia is an aggressive malignancy with high morbidity and mortality, and chemotherapy is the primary treatment modality. However, chemotherapy often induces neutropenia (chemotherapy-induced neutropenia, CIN), increasing the risk of infectious complications and mortality. Current research suggests that gut microbiota may play a significant role in chemotherapy’s efficacy and side effects.

**Objective:**

This study aimed to investigate whether gut microbiota can predict the duration of chemotherapy-induced neutropenia in leukemia patients.

**Methods:**

We included 56 leukemia patients from the Hematology Department of the Second Affiliated Hospital of Nanchang University, collecting fecal samples 1 day before and 1 day after chemotherapy. The diversity and community structure of gut microbiota were analyzed using 16S rRNA gene sequencing. Patients were divided into two groups based on the duration of neutropenia post-chemotherapy: Neutropenia ≤7 Days Group (NLE7 Group) and Neutropenia > 7 Days Group (NGT7 Group). Comparative analysis identified characteristic microbiota.

**Results:**

After chemotherapy, gut microbiota diversity significantly decreased (*p* < 0.05). In the NGT7 Group, the relative abundance of *Enterococcus* before chemotherapy was significantly higher than in the NLE7 Group (*p* < 0.05). ROC curve analysis showed that the relative abundance of *Enterococcus* had high predictive accuracy for the duration of neutropenia (AUC = 0.800, 95% CI: 0.651–0.949).

**Conclusion:**

The abundance of *Enterococcus* before chemotherapy can predict the duration of chemotherapy-induced neutropenia. These findings provide new evidence for gut microbiota as a predictive biomarker for chemotherapy side effects and may guide personalized treatment for leukemia patients.

## 1 Introduction

Acute leukemia is a highly aggressive malignancy with both high incidence and mortality rates, characterized by the clonal proliferation of bone marrow progenitor cells with a blockade in differentiation. The standard treatment for leukemia typically involves intensive chemotherapy with cytarabine and anthracycline (the 7 + 3 regimen) and hypomethylating agents (Liu, 2021). However, this type of chemotherapy significantly affects multiple normal, rapidly dividing cells. In particular, when chemotherapy leads to the depletion of hematopoietic progenitor cells, acute bone marrow suppression may occur. At this point, hematopoietic stem cells initiate self-renewal and proliferate to form progenitor cells in order to maintain the homeostasis of the hematopoietic system (China Anti-Cancer Association Tumor Clinical Chemotherapy Professional Committee and China Anti-Cancer Association Tumor Support Therapy Professional Committee, 2023). Several studies have demonstrated that the degree and duration of chemotherapy-induced neutropenia (CIN) are strongly correlated with the risk of infectious complications and mortality. Notably, the occurrence of febrile neutropenia (FN) further exacerbates the risks, leading to potential reductions in chemotherapy dosage and/or delays in treatment, and in severe cases, even death (Bodey et al., 1966; Lalami and Klastersky, 2017). Factors such as poor physical condition, suboptimal nutritional status, low baseline, nadir blood cell counts during the first chemotherapy cycle, and high-dose chemotherapy have been identified as key predictors of CIN (Lyman et al., 2005). Additionally, patient comorbidities, age, individual pharmacokinetics and pharmacodynamics also influence the occurrence of CIN (Kloft et al., 2006; Stabell et al., 2008). Nonetheless, these factors only partially explain the variation among patients. Despite receiving the same chemotherapy, there are significant differences in the frequency, severity, and duration of neutropenia among patients (Stabell et al., 2008). Therefore, identifying the underlying causes and risk factors of chemotherapy-induced neutropenia is crucial.

The gut microbiota, comprising bacteria, archaea, and eukaryotes organisms residing in the gastrointestinal tract, is a critical component of the human body’s complex micro-ecosystem, interacting with the host to influence various physiological processes and pathological conditions (O'Toole and Paoli, 2023; Caballero-Flores et al., 2023). The connection between microbiota and tumours has garnered significant attention in recent years. Studies have shown that conventional treatments, including radiotherapy, chemotherapy, and immunotherapy, can alter the gut microbiota composition. Furthermore, the composition of the microbiota and specific bacterial species can influence these treatments’ efficacy and side effects by modulating the inflammatory response and immune system (Andreeva et al., 2020; Cheng et al., 2020). For example, the chemotherapy drug irinotecan, while effective in killing cancer cells, also damages normal intestinal epithelial cells and symbiotic microorganisms, leading to gastrointestinal toxicity and microbiota dysbiosis. Additionally, β-glucuronidase secreted by gut bacteria prolongs irinotecan’s clearance time in the body, exacerbating its gastrointestinal toxicity (Yue et al., 2021). Recent studies by Yoon et al. reported that in a cohort of newly diagnosed diffuse large B-cell lymphoma patients, the abundance of Enterobacteriaceae was associated with febrile neutropenia and poor survival following R-CHOP chemotherapy (Yoon et al., 2023). Similarly, Galloway-Peña et al. demonstrated that baseline microbiome diversity is a strong independent predictor of infection during induction chemotherapy in acute myeloid leukemia patients, with higher levels of Porphyromonadaceae seeming to protect against infections (Galloway-Peña et al., 2020). Moreover, the gut microbiota plays a key role in normal hematopoiesis and may affect the white blood cell counts through hematopoietic function (Staffas et al., 2018). For instance, long-term antibiotic use, which alters the gut microbiota, has been linked to hematological side effects, including anemia and neutropenia. Mouse studies have shown that antibiotic-induced microbiota depletion and bone marrow suppression are due to a lack of stable microbial products, which circulate in the blood and promote hematopoiesis through basal inflammatory signalling. These mechanisms are particularly relevant for patients undergoing long-term antibiotic treatment or recovering from hematopoietic stem cell transplants (Galloway-Peña et al., 2020). These studies highlight the close relationship between the gut microbiota, the hematopoietic system, and chemotherapy-related complications.

Existing studies primarily focus on the relationship between the gut microbiota and the incidence, severity, and frequency of bone marrow suppression, as well as its association with FN after chemotherapy. The innovation of this study lies in exploring the predictive role of the pre-chemotherapy gut microbiota in determining the duration of bone marrow suppression. Based on the risk stratification of patients with febrile neutropenia, those with neutropenia lasting longer than 7 days are classified as high-risk (Galloway-Peña et al., 2020). Therefore, this study stratifies patients into two groups based on the duration of neutropenia (7 days) and utilizes microbiome sequencing technology to compare the diversity and community structure of gut microbiota before and after chemotherapy in leukemia patients. Characteristic bacterial taxa are identified in both groups. Predicting the duration of neutropenia can help optimize patient management, prevent infectious complications, and facilitate the rational use of antibiotics and granulocyte colony-stimulating factor (G-CSF). Furthermore, the results of this study may provide new insights for subsequent research into microbiome-based therapies aimed at reducing chemotherapy-induced bone marrow suppression.

## 2 Materials and methods

### 2.1 Study patients and fecal sample collection

This study is a retrospective analysis involving 56 newly diagnosed leukemia patients treated in the Hematology Department of the Second Affiliated Hospital of Nanchang University between 2023 and 2024. To be eligible, patients had to meet the following inclusion criteria: (i) age > 18 years, (ii) newly diagnosed with leukemia according to the WHO classification of myeloid neoplasms, and (iii) no gastrointestinal surgery or antitumor treatment within 6 months before enrollment. The exclusion criteria were: (i) patients with acute promyelocytic leukemia, (ii) those with other gastrointestinal diseases, (iii) those who had previously received antitumor treatment, (iv) those with other tumours, and (v) patients who had used antibiotics and/or probiotics within 90 days or had received nasal tube feeding or parenteral nutrition during the study period. After obtaining informed consent, fecal samples (5-10 g) were collected from patients 1 day before the first chemotherapy and on the first day after chemotherapy. A total of 47 pre-chemotherapy and 34 post-chemotherapy fecal samples were collected. All samples were immediately frozen and transported to the laboratory within 24 h using cold-chain logistics. Basic information and baseline clinical data at admission were obtained from the hospital’s case management system.

### 2.2 DNA extraction and library construction

DNA was extracted from fecal samples using the Advanced Stool DNA Kit. PCR amplification of the 16S rRNA V3-V4 region of standard bacteria was performed using primers (human: 341F-806R, 341F 5′-CCTAYGGGRBGCASCAG-3′ and 806R 5′-GGACTACNNGGGTWTCTAAT-3′).

PCR reactions were carried out on a BioRad S1000 thermal cycler (Bio-Rad Laboratory, CA, USA) with the following cycling conditions: 94°C for 5 min (initial denaturation), followed by 30 cycles of 94°C for 30 s, 57°C for 30 s, and 72°C for 30 s, with a final extension at 72°C for 10 min and storage at 4°C. PCR products from the same sample were pooled. The fragment size and length of PCR products were verified using 1% agarose gel electrophoresis. Concentrations were compared using GeneTools Analysis Software (Version 4.03.05.0, SynGene), and PCR products were mixed in appropriate ratios according to sequencing volume requirements.

PCR products were purified using the E.Z.N.A.® Gel Extraction Kit (Omega, USA) and eluted with TE buffer. Libraries were constructed following the standard protocol of the NEBNext® Ultra™ II DNA Library Prep Kit for Illumina® (New England Biolabs, USA). The constructed amplicon libraries were sequenced on an Illumina Nova 6,000 platform for PE250 sequencing (Guangdong Magigene Biotechnology Co., Ltd., Guangzhou, China).

### 2.3 Sequencing data bioinformatics analysis

#### 2.3.1 Analysis of raw date

The raw sequencing data were assembled and subjected to quality control to ensure accuracy and optimization. The samples were then distinguished, followed by operational taxonomic unit (OTU) clustering and species classification analysis. Based on the OTUs, various diversity indices were further analyzed. Additionally, multivariate analyses, significance testing, and a series of statistical and visual analyses were conducted to examine the community composition and phylogenetic information of multiple species.

#### 2.3.2 Community composition analysis

OTU table sequences were categorized at five taxonomic levels: phylum, class, order, family, and genus. Relative abundance was calculated for each taxon and represented by community bar charts. Venn diagrams were used to analyze shared and unique OTUs in the gut microbiota.

#### 2.3.3 Alfa diversity and beta diversity analysis

To assess the ecological diversity and structure of the fecal microbiota, alfa diversity was calculated using the Chao richness index, Ace index, Shannon index, and Simpson index. Beta diversity was evaluated by performing principal coordinates analysis (PCoA) based on the weighted UniFrac distance algorithm, allowing for comparison of microbial diversity across all samples.

#### 2.3.4 Species difference analysis

Based on the community abundance data, differences in microbial community abundance were detected. Linear discriminant analysis (LDA) was used to estimate the impact of species abundance differences, followed by the Mann–Whitney U test to identify significantly different taxa.

### 2.4 Statistical analysis

Continuous data were presented as mean ± standard deviation (SD) and compared between groups using an independent sample t-test. Categorical data were expressed as n (%) and compared using the chi-square test or Fisher’s exact test. A *p*-value <0.05 was considered statistically significant. ACE, Chao1, Shannon, and Simpson indices represented the Alpha diversity of gut microbiota. Beta diversity was compared using principal coordinate analysis (PCoA) and analysis of similarities (ANOSIM). Linear discriminant analysis (LDA) and effect size (LEfSe) were used to analyze the relative abundance of different taxa. Discriminatory features with a log LDA score > 2 and Kruskal-Wallis *p* < 0.05 were identified. The Spearman correlation was used to analyze the relationship between gut microbiota and clinical indicators. ROC curves were plotted to evaluate the predictive value of gut microbiota features for neutropenia duration greater than 7 days, and the area under the curve (AUC) was calculated. All significant tests were two-sided, with p < 0.05 or adjusted p < 0.05 considered statistically significant.

## 3 Results

### 3.1 Baseline characteristics

This study included 56 leukemia patients diagnosed at the Hematology Department of the Second Affiliated Hospital of Nanchang University. A total of 81 fecal samples were collected, with 47 samples before and 34 samples after chemotherapy. Due to some patients being discharged early, only 37 patients had complete clinical data for assessing the duration of grade 4 myelosuppression after chemotherapy. Based on this, patients were divided into the Neutropenia ≤7 Days Group (NLE7 Group) and the Neutropenia >7 Days Group (NGT7 Group). The clinical characteristics of the 56 patients are summarized in Table 1.

**Table 1:** Clinical characteristics of the 56 leukemia patients.

Patient characteristic	Total (n = 56)	NLE7 Group (n = 15 )	NGT7 Group (n = 22)
**Age, median years, mean (±SD)**	62.34 ± 13.64	63 ± 15.4	59.5 ± 12.4
**Sex, man, n (%)**	32 (57.1%)	9 (60%)	11 (50%)
**Weight, mean (±SD)**	60.2 ± 8.3	60.0 ± 9.2	61.0 ± 8.3
**AML phenotype, n (%)**			
M0	3 (5.4%)	1 (6.7%)	0 (0%)
M1	10 (17.9%)	0 (0%)	7 (31.8%)
M2	18 (32.1%)	4 (26.7%)	5 (22.7%)
M4	17 (30.4%)	7 (46.7%)	6 (27.3%)
M5	8 (14.3%)	3 (20%)	4 (18.2%)
**Risk categorization, n (%)**			
Favourable	26 (57.1%)	8 (53.3%)	13 (59.1%)
Intermediate	16 (28.6%)	3 (20%)	5 (22.7%)
Adverse	12 (21.4%)	4 (26.7%)	4 (18.2%)
**Chemotherapy regimens, n (%)**			
7&3	32 (57.1%)	5 (33.3%)	14 (63.6%)
Ven + HMA	20 (35.7%)	9 (60%)	5 (22.7%)
Others	4 (7.1%)	1 (6.7%)	3 (13.6%)
**Baseline leukocyte count mean (±SD)**	57.74 ± 63.67	48.41 ± 53.90	60.30 ± 61.80
**Baseline platelet count mean (±SD)**	52.75 ± 65.48	50.6 ± 57.52	67.78 ± 76.33
**Baseline Hemoglobin count mean (±SD)**	75.51 ± 22.90	86 ± 28.42	77.45 ± 28.42
**Duration of neutropenia (days)**		3 ± 2.5	20.4 ± 11.0

NLE7, Neutropenia ≤7 Days; NGT7, Neutropenia >7 Days;M0, Acute Myeloblastic Leukemia (without maturation); M1, Acute Myeloblastic Leukemia (with minimal maturation); M2, Acute Myeloblastic Leukemia (with maturation); M3, Acute Promyelocytic Leukemia; M4, Acute Myelomonocytic Leukemia; M5, Acute Monocytic Leukemia; 7&3, Cytarabine + Daunorubicin; Others, Decitabine+Cytarabine, Azacitidine+Cytarabine; SD, standard deviation.

### 3.2 Changes in gut microbiota diversity and composition before and after chemotherapy

**Figure 1:**
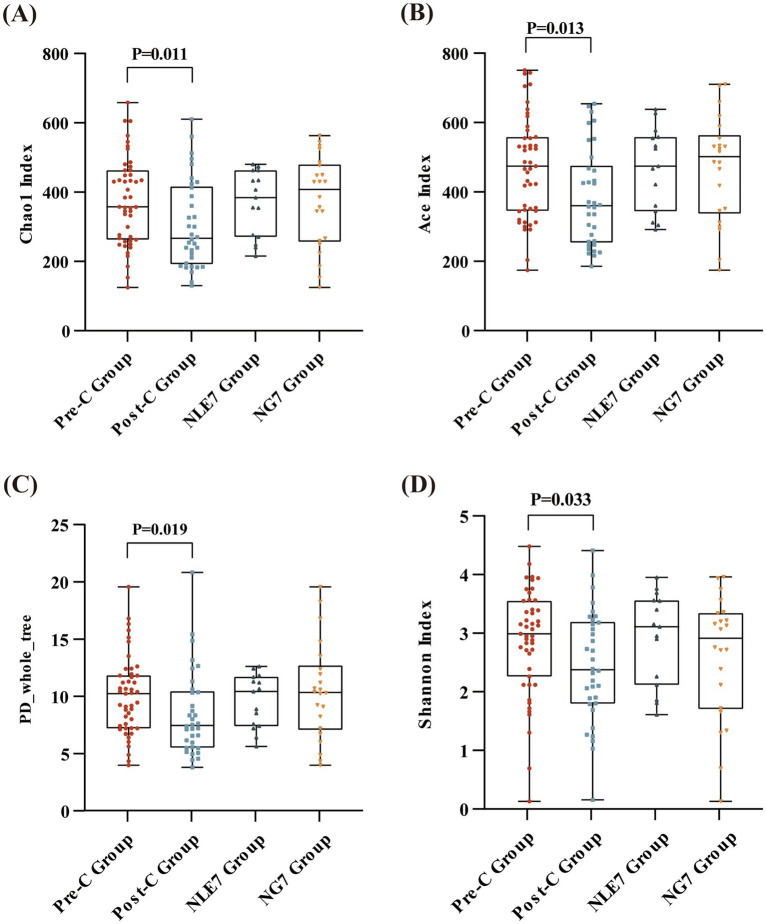
Alpha diversity at the OTU level (measured in terms of the **(A)** Chao1 Index, **(B)** Ace Index, **(C)** PD_whole_tree Index, **(D)** and Shannon index) between the Pre-C Group (*n* = 47) and Post-C Group (*n* = 36), and the NLE7 Group (*n* = 47) and NG7 Group (*n* = 36). Differences across groups were compared using the Wilcoxon rank-sum test. *p* < 0.05 was considered statistically significant.

**Figure 2:**
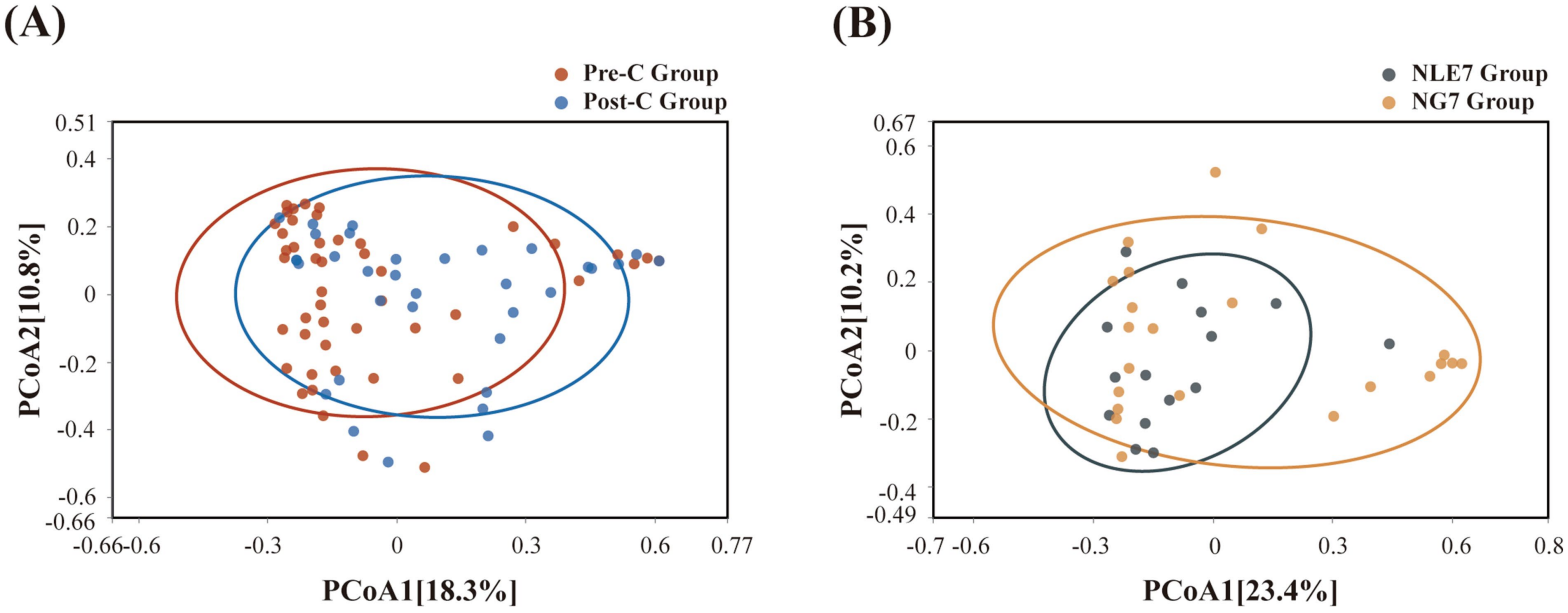
**(A)** Beta diversity was evaluated by Principal coordinate analysis of Bray-Curtis between the Pre-C Group and Post-C Group, and **(B)** the NLE7 Group and NG7 Group.

**Figure 3:**
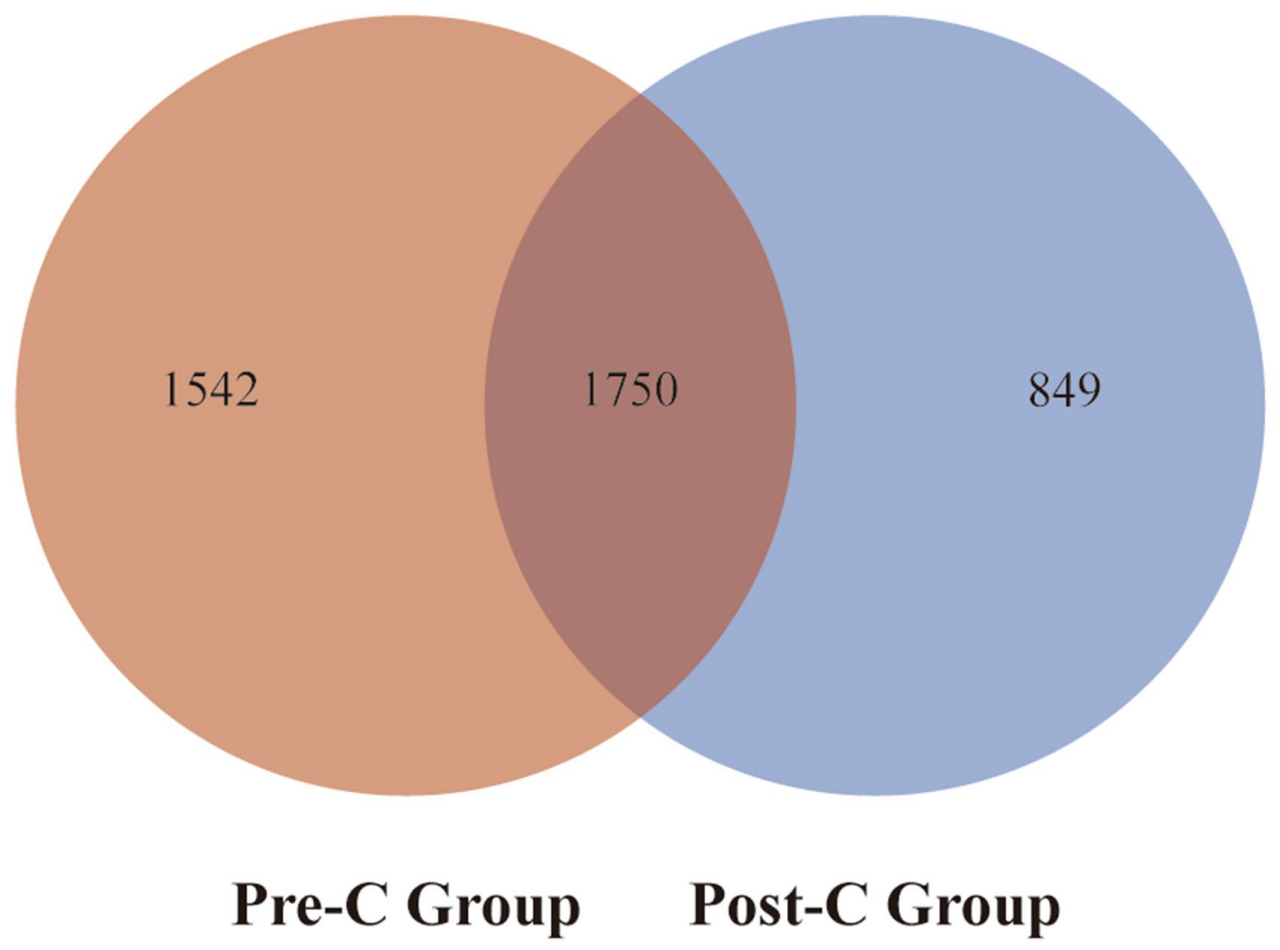
The Venn diagram of the total gut bacteria at the OTUs level for the Pre-C Group and the Post-C Group. Red represents the Pre-C Group, blue refers to the Post-C Group, and the overlapping part stands for the number of common bacteria at the OTUs level in the two groups.

The 81 fecal samples were diided into the Pre-Chemotherapy Group (Pre-C Group) and the Post-Chemotherapy Group (Post-C Group) based on collection time. Based on 16S rRNA gene sequencing analysis, the microbial diversity and composition of the two groups were further statistically analyzed. OTU abundance represented richness, while alpha diversity was represented by Chao1, ACE, Shannon, and Simpson indices. At the OTU level, both alpha and beta diversity indices are shown in Figures 1A–D. According to the Wilcoxon rank-sum test, alpha diversity significantly decreased post-chemotherapy compared to pre-chemotherapy (*p* = 0.011 [Chao1], 0.013 [ACE], 0.019 [PD_whole_tree], 0.033 [Shannon]). Although principal coordinate analysis (PCoA) based on the weighted UniFrac distance algorithm showed statistically significant differences between pre-chemotherapy and post-chemotherapy samples (ANOSIM R = 0.035, *p* = 0.041), the overall gut microbiota structure was not significantly separated (Figure 2A). The Venn diagram showed 1750 shared OTUs between the two groups, with 1,542 unique OTUs in the Pre-C group and 849 in the Post-C group (Figure 3). These results indicate a significant reduction in gut microbiota diversity after chemotherapy, with no significant change in community structure composition.

**Figure 4:**
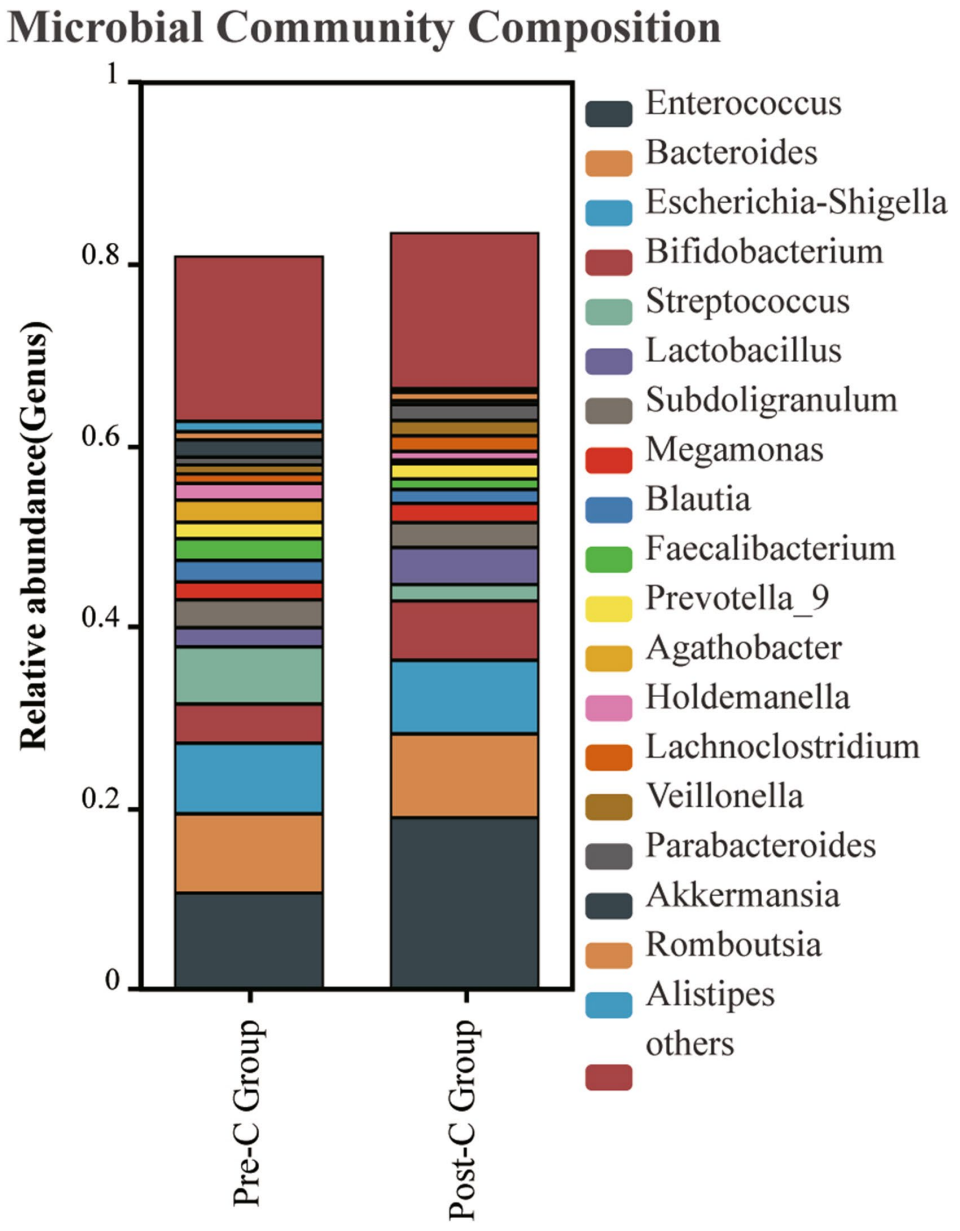
The gut bacteria composition at the genus level between the Pre-C Group and the Post-C Group was plotted. The abscissa is the group name, and the ordinate represents the relative abundance of bacteria in the sample. Different colors represent different bacterial genus, and the length of the column represents the proportion of bacterial groups.

In the taxonomic comparison of gut microbiota composition at the class level, it was found that the baseline abundance of *Clostridia* (P: 0.012) and *Saccharimonadia* (P: 0.004) in the Post-C group was significantly lower than in the Pre-C group. At the order level, the abundance of *Clostridiales* and *Saccharimonadales* was significantly reduced post-chemotherapy (P: 0.023). At the family level, *Lachnospiraceae* decreased (P: 0.0433). Outside the top 20 in abundance, *Atopobiaceae* and *Saccharimonadaceae* also decreased. At the genus level, the top five bacteria in both groups were *Enterococcus*, *Bacteroides*, *Escherichia-Shigella*, *Bifidobacterium*, and *Streptococcus*, with no significant species differences observed (Figure 4).

### 3.3 Gut microbiota differences in NLE7 group and NGT7 group before chemotherapy

This study collected pre-chemotherapy fecal samples from 37 leukemia patients and assessed the duration of grade 4 myelosuppression. Patients were divided into two groups based on a seven-day threshold. The gut microbiota in pre-chemotherapy samples of both groups was compared using 16S rRNA gene sequencing analysis. The NLE7 Group had lower Chao1 and ACE indices, and higher Shannon and Simpson indices compared to the NGT7 Group, but the differences were not statistically significant (Figures 1A–D). Beta diversity comparison using the Bray-Curtis algorithm and distance matrix analysis with ANOSIM indicated that inter-group differences were greater than intra-group differences but not significant (R = 0.056, *p* = 9.5e-02) (Figure 2B), suggesting no significant differences in the species number, diversity, and community structure of microbiota between the two groups.

**Figure 5:**
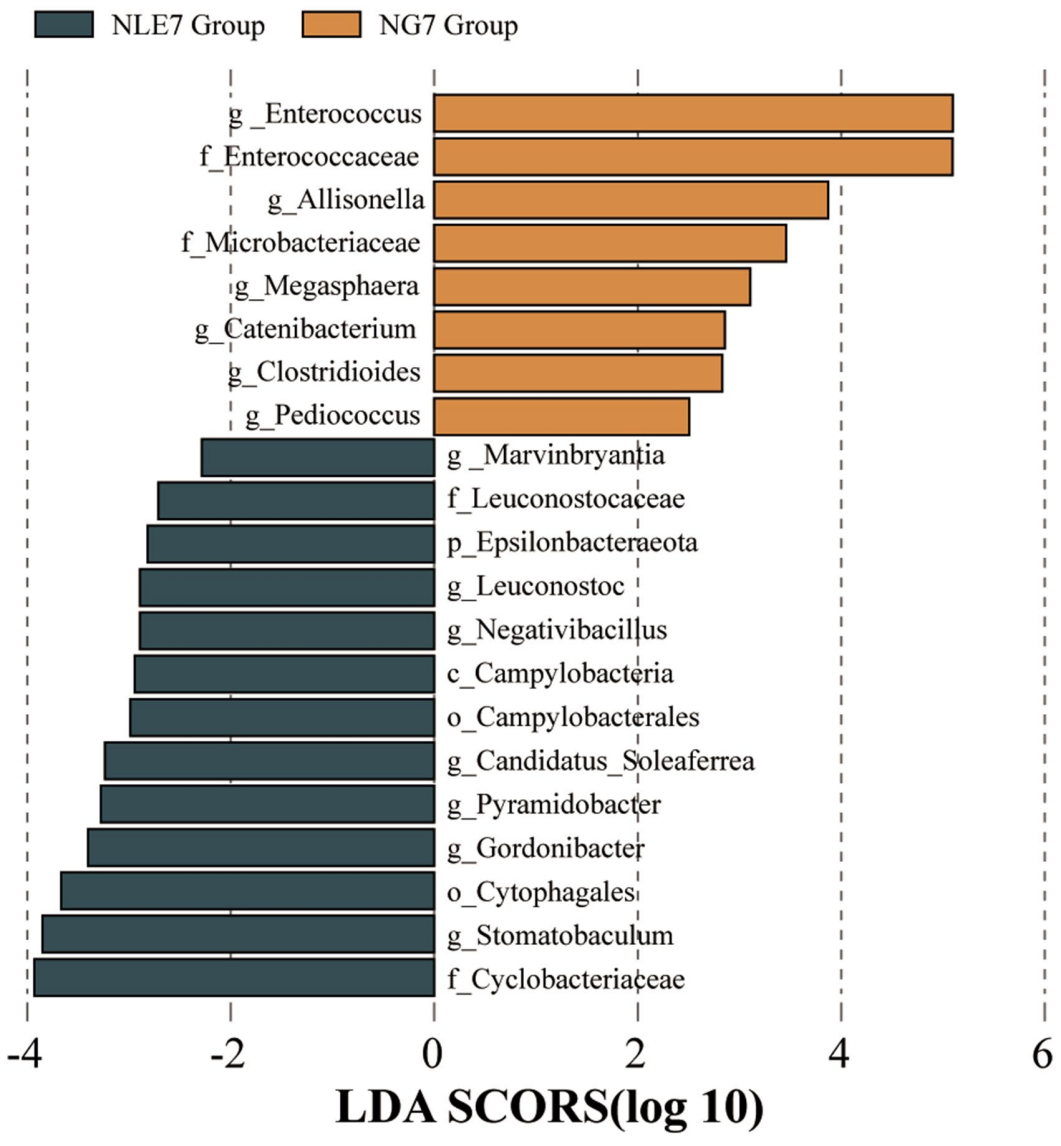
(1)The bar chart of Linear Discriminant Analysis (LDA) scores shows the taxa with the most significant differences between the NLE7 Group and NGT7 Group. Only taxa with an LDA score > 2 are shown.

To further identify significantly different abundant microbiota between the groups, LEfSe analysis with an LDA score (Log10) = 2 was used to screen important microbial biomarkers. The NLE7 Group had characteristic species, mainly at the class level (*Campylobacteria*), order level (*Cytophagales*, *Campylobacterales*), family level (*Cyclobacteriaceae*, *Leuconostocaceae*), and genus level (*Stomatobaculum*). The NGT7 Group had significantly different species mainly at the phylum level (*Epsilonbacteraeota*), family level (*Enterococcaceae*, *Mycobacteriaceae*), and genus level (*Enterococcus*) (Figure 5).

Based on the LEfSe results, further analysis of the community distribution and verification of relative abundance differences between the two groups was conducted using the Mann–Whitney U test. Analysis at the phylum level showed that the main phyla in both groups were *Firmicutes*, *Bacteroidetes*, *Proteobacteria*, *Actinobacteria*, and *Verrucomicrobia*. Among these, differences in *Firmicutes* and *Bacteroidetes* were not significant. However, *Proteobacteria* had a lower mean relative abundance in the NLE7 Group (5.78%) compared to the NGT7 Group (13.04%). *Actinobacteria* had a higher mean relative abundance in the NLE7 Group (8.17%) compared to the NGT7 Group (4.36%), but the differences were not statistically significant. At the family level, the top three families were *Lachnospiraceae*, *Enterococcaceae*, and *Ruminococcaceae*. *Enterococcaceae* had a significantly lower mean relative abundance in the NLE7 Group (3.38%) compared to the NGT7 Group (21.25%) (*p* = 0.002). At the genus level, *Enterococcus* showed a significant difference in relative abundance between the two groups (*p* = 0.02).

### 3.4 Correlation between gut microbiota, clinical characteristics, and neutropenia duration

**Figure 6:**
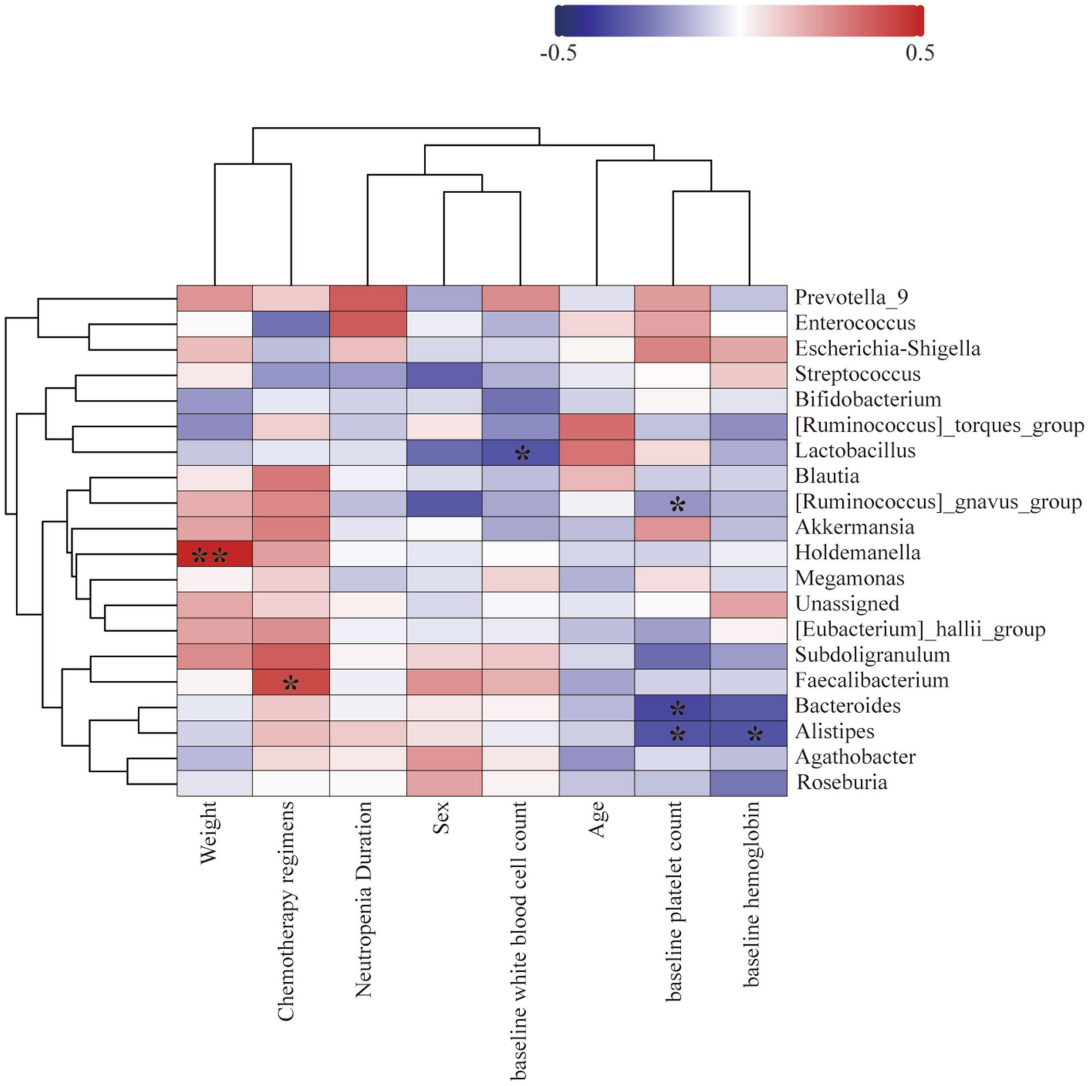
The heatmap illustrates the correlation between different bacteria and patient age, gender, weight, baseline white blood cell count, baseline hemoglobin, baseline platelet count, and the duration of neutropenia. Blue squares represent negative Spearman rank correlation coefficients, while red squares represent positive Spearman rank correlation coefficients. Darker shades of blue or red indicate higher correlation values. Pearson’s correlation coefficients are shown in the figure (* *p* < 0.05, ** *p* < 0.01).

To determine the correlation between gut microbiota, clinical characteristics, and neutropenia duration, we collected data on age at diagnosis, weight, Chemotherapy regimens, baseline white blood cell count, baseline hemoglobin, and baseline platelet count from 37 AML patients. These clinical characteristics did not show significant correlations with neutropenia duration post-chemotherapy. Among the top 20 most abundant bacterial families, post-chemotherapy neutropenia duration was positively correlated with *Enterococcaceae* (r = 0.370, *p* = 0.024). Patient weight was positively correlated with *Holdemanella* (r = 0.479, *p* = 0.003); baseline white blood cell count was negatively correlated with *Lactobacillaceae* (r = −0.337, *p* = 0.42); platelet count was negatively correlated with *Alistipes* (r = −0.343, *p* = 0.036) *Bacteroides* (r = −0.363, *p* = 0.027) and *Ruminococcus_gnavus_group* (r = −0.349, *p* = 0.034), hemoglobin was negatively correlated with *Alistipes* (r = −0.347, *p* = 0.035) (Figure 6).

### 3.5 Predictive role of *Enterococcus*

**Figure 7:**
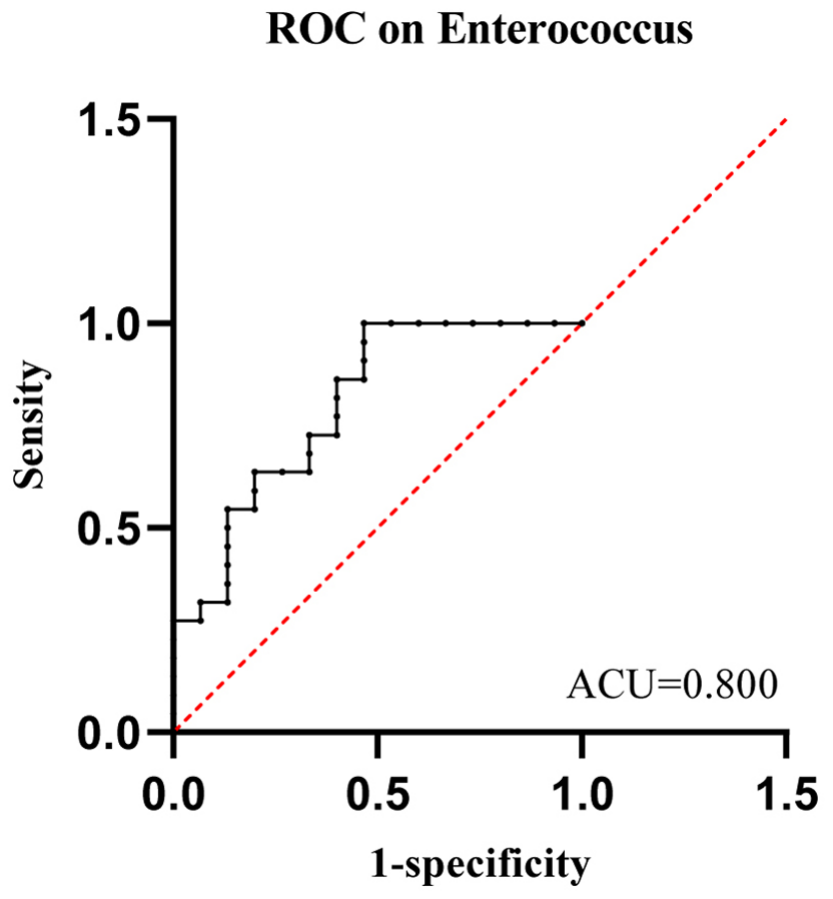
The ROC curve demonstrates the predictive value of Enterococcaceae for outcomes in the NLE7 Group and NGT7 Group.

We evaluated the potential of gut microbiota as a biomarker to predict whether neutropenia lasts more than 7 days. ROC curve analysis showed that the relative abundance of *Enterococcaceae* at baseline had strong predictive power for whether leukemia patients would experience neutropenia lasting more than 7 days post-chemotherapy (AUC = 0.800; 95% CI: 0.651–0.949; *p* = 0.002). The abundance threshold for *Enterococcaceae* was 22%, with a sensitivity of 53% and a specificity of 100% (Figure 7). Based on this abundance threshold, patients were divided into two groups. Binary logistic regression analysis showed significant differences between the two groups [*p* = 0.024, EEP(B) = 1.246]. Overall, a relative abundance of *Enterococcaceae* > 22% can predict grade 4 myelosuppression >7 days post-chemotherapy, validating *Enterococcus* as a predictive role.

## 4 Discussion

Increasing research has focused on the relationship between gut microbiota and chemotherapy-induced toxicity and related complications in cancer treatment. However, determining the specific connection between these factors remains challenging. Few studies have directly explored the relationship between gut microbiota characteristics in leukemia patients and the duration of neutropenia following chemotherapy. In our study, leukemia patients were divided into two groups based on a seven-day threshold for the duration of post-chemotherapy neutropenia. Comparing the diversity and community structure of gut microbiota before chemotherapy, there were no significant differences between the two groups. However, the differential species analysis revealed that the species primarily characterizing the <7 days group included *Campylobacteria*, *Cytophagales*, *Campylobacterales*, *Cyclobacteriaceae*, *Leuconostocaceae*, and *Stomatobaculum*. In contrast, the ≥7 days group was characterized by *Epsilonbacteraeota*, *Enterococcaceae*, and *Mycrobacteriaceae*. Correlation analysis showed that *Enterococcaceae* was associated with the duration of neutropenia, suggesting its predictive value. Additionally, our study confirmed that both the alpha diversity and beta diversity of gut microbiota in AML patients decreased after chemotherapy, consistent with previous extensive research (Montassier et al., 2015).

The correlation between gut microbiota and chemotherapy has been extensively studied. Gut microbiota can regulate chemotherapeutic drugs through various mechanisms, including direct cytotoxicity, bacterial translocation, immune response, drug metabolism, drug efficacy, and the elimination and impairment of anti-cancer effects (Yip and Chan, 2015). On the other hand, chemotherapy significantly reduces gut microbial diversity, even without antibiotic use, and our findings corroborate this (Lee et al., 2019). In addition, studies have demonstrated the association between gut microbiota and hematopoiesis. For example, Staffas et al. utilized a germ-free mouse model of HSCT. They found that providing additional enteral nutrition to the germ-free mice improved gut microbiota depletion, thereby promoting hematopoietic recovery (Staffas et al., 2018). Hao Guo et al. discovered that the increased abundance of *Muribaculaceae* and *Enterococcaceae* was associated with hematopoietic recovery and gastrointestinal repair after radiotherapy (Guo et al., 2020).

*Enterococcus* is a genus of Gram-positive, facultative anaerobic cocci commonly found in the gastrointestinal tracts of humans and animals. In healthy individuals, *Enterococcus* acts as a commensal organism, maintaining gut microbial balance and regulating the immune system. However, it is also considered an opportunistic pathogen, especially in immunocompromised individuals, where it is more likely to cause infections. Multiple studies have linked *Enterococcus* to adverse events. For instance, in children with ALL, the dominance of *Enterococcus* in the gut predicts FN or diarrheal diseases post-chemotherapy (Hakim et al., 2018). Sørum et al. recently explored the connection between gut microbiota composition and the duration of neutropenia, finding that prolonged neutropenia in children with acute lymphoblastic leukemia post-chemotherapy was associated with reduced abundances of *Ruminococcaceae* and *Lachnospiraceae*, as well as a decrease in *Veillonella* and an overgrowth of *Enterococcus* (Sørum et al., 2024), which is consistent with our conclusion. The *Enterococcus* can trigger a systemic inflammatory response (Yuen and Ausubel, 2014), releasing pro-inflammatory cytokines (Park et al., 2013), which attract neutrophils to migrate to extravascular tissues. Combined with decreased bone marrow function, this can reduce peripheral blood ANC, thereby prolonging the duration of neutropenia post-chemotherapy. Additionally, studies have shown that a high abundance of *Enterococcus* prior to chemotherapy predicts an increased risk of infection during the neutropenic phase post-chemotherapy (Hakim et al., 2018), leading to an increased use of antibiotics. Antibiotic use alters the microbiome composition and metabolites, which in turn impacts hematopoietic homeostasis and increases the likelihood of neutropenia (Fernandez-Sanchez et al., 2024). Chen et al. explored the relationship between granulopoiesis and gut microbiota in a chemotherapy-induced neutropenic mouse model and found that gut decontamination with oral antibiotics suppressed T-cell production of IL-17A and impaired neutrophil recovery (Chen et al., 2022). Josefsdottir et al. demonstrated that broad-spectrum antibiotic treatment-induced microbiota depletion disrupted baseline Stat1 signalling and altered T cell homeostasis, leading to impaired granulocyte maturation (Josefsdottir et al., 2017). Based on these findings, we hypothesize that a high abundance of *Enterococcus* prior to chemotherapy may indirectly prolong the time required for granulopoiesis. The relationship and underlying mechanisms between *Enterococcus* and prolonged neutropenia post-chemotherapy warrant further investigation.

An important contribution of our study is the identification of *Enterococcus* as a potential biomarker for predicting the duration of chemotherapy-induced neutropenia in leukemia patients. ROC curve analysis showed that the relative abundance of *Enterococcus* had a high accuracy in predicting the duration of neutropenia, suggesting new possibilities for utilizing gut microbiota in personalized treatment in future clinical practice. Additionally, given the increasing preclinical evidence that modulation of gut microbiota can mitigate chemotherapy-related complications, translating our findings into clinical practice could represent a significant breakthrough in preventing such complications. For example, supplementing specific probiotics may help maintain or restore the healthy balance of gut microbiota, thereby reducing chemotherapy-induced neutropenia and other complications (Chrysostomou et al., 2023). However, it is important to note that with the increasing use of probiotics, there have been more reports on the incidence of bacteremia associated with probiotic use in both immunocompetent and immunocompromised patients (Sada et al., 2024). Therefore, probiotics must be used cautiously, and more clinical evidence is needed to guide their safe application.

This study has some limitations. Firstly, the sample size is relatively small, and not all patients provided stool samples after chemotherapy, which may limit the statistical power and generalizability of the findings. Additionally, it did not account for other factors that could contribute to differences in the gut microbiota, such as diet, age, and body mass index. Future studies with larger sample sizes may improve our ability to detect inter-group differences and minimize the impact of these factors through subgroup analyses. Additionally, although we found an association between the abundance of *Enterococcus* and the duration of neutropenia, the specific mechanisms remain unclear and require further experimental investigation.

Overall, our study reveals the potential role of gut microbiota, particularly *Enterococcus*, in predicting the duration of chemotherapy-induced neutropenia in leukemia patients. This finding provides new evidence for gut microbiota as predictive biomarkers for chemotherapy side effects and may offer important guidance for the personalized treatment of leukemia patients. Future research should further explore the regulatory mechanisms of gut microbiota and validate our results in larger-scale clinical trials to facilitate the clinical translation and application of these findings.

## 5 Conclusion

Our study confirmed the association between gut microbiota and the duration of neutropenia following chemotherapy in leukemia patients. Given the increasing preclinical evidence that modulating gut microbiota can mitigate chemotherapy-related complications, translating these findings into clinical practice could represent a significant breakthrough in preventing such complications.

## Data Availability

The data presented in this study are deposited in the NCBI SRA repository, accession number PRJNA1233767.
